# Cognitive and oculomotor performance in subjects with low and high schizotypy: implications for translational drug development studies

**DOI:** 10.1038/tp.2016.64

**Published:** 2016-05-17

**Authors:** I Koychev, D Joyce, E Barkus, U Ettinger, A Schmechtig, C T Dourish, G R Dawson, K J Craig, J F W Deakin

**Affiliations:** 1 Department of Community-Based Psychiatry, Neuroscience and Psychiatry Unit, The University of Manchester, School of Community-Based Medicine, Manchester, UK; 2 Department of Psychiatry, University of Oxford, Warneford Hospital, Oxford, UK; 3 Cognition, Schizophrenia and Imaging Lab, Department of Psychosis Studies, Institute of Psychiatry, Psychology and Neuroscience, Denmark Hill, London; 4 Department of Psychology, University of Wollongong, Wollongong, New South Wales, Australia; 5 Department of Psychiatry, School of Community-Based Medicine, The University of Manchester, Manchester, UK; 6 Department of Psychology, University of Bonn, Bonn, Germany; 7 Department of Neuroimaging, Institute of Psychiatry, King's College London, London, UK; 8 P1vital, Manor House, Howbery Park, Wallingford, UK

## Abstract

The development of drugs to improve cognition in patients with schizophrenia is a major unmet clinical need. A number of promising compounds failed in recent clinical trials, a pattern linked to poor translation between preclinical and clinical stages of drug development. Seeking proof of efficacy in early Phase 1 studies in surrogate patient populations (for example, high schizotypy individuals where subtle cognitive impairment is present) has been suggested as a strategy to reduce attrition in the later stages of drug development. However, there is little agreement regarding the pattern of distribution of schizotypal features in the general population, creating uncertainty regarding the optimal control group that should be included in prospective trials. We aimed to address this question by comparing the performance of groups derived from the general population with low, average and high schizotypy scores over a range of cognitive and oculomotor tasks. We found that tasks dependent on frontal inhibitory mechanisms (*N*-Back working memory and anti-saccade oculomotor tasks), as well as a smooth-pursuit oculomotor task were sensitive to differences in the schizotypy phenotype. In these tasks the cognitive performance of ‘low schizotypes' was significantly different from ‘high schizotypes' with ‘average schizotypes' having an intermediate performance. These results indicate that for evaluating putative cognition enhancers for treating schizophrenia in early-drug development studies the maximum schizotypy effect would be achieved using a design that compares low and high schizotypes.

## Introduction

Cognitive impairment is a core symptom of schizophrenia that predicts functional outcome^1, 2, 3^ and treatment adherence.^4^ However, it is largely unaffected by currently available medication^5^ and the development of drugs to treat cognitive deficits in schizophrenia is a recognised unmet need.^6^

Although a number of putative cognitive enhancers have been examined in trials none have been approved for treatment.^7^ Lack of clinical efficacy was the main reason for attrition revealing a critical gap in translating efficacy evidence from animal models to patients. One strategy to address this is to assess efficacy at Phase 1 of drug development and discontinue development if no efficacy is demonstrated thus saving time and resources from Phase 2 and 3 trials.^8^ Such experimental or translational medicine studies use biomarkers of the proposed core pathophysiology as proxies of efficacy rather than traditional clinical end-points to shorten the time needed to reach ‘Go/No-Go' decisions.^9^ This approach can be complemented by selecting either patients who are more likely to respond (mild symptomatology or medication-free) or subclinical healthy volunteers that lack the confounds of patient groups (for example, heterogeneous symptom and medication profiles).

In the case of schizophrenia, early assessment of cognitive enhancers efficacy can be sought in surrogate patient populations such as individuals with high levels of schizotypy.^10, 11, 12^ Schizotypy is a term coined by Meehl^13^ under the influence of Rado^14^ and is used to describe a latent personality structure associated with schizophrenia risk with the affected individuals referred to as ‘schizotypes'.^13^ The severity of these features range along a continuum that funnels into overt psychosis at its extreme.^15, 16, 17^ Schizophrenia and schizotypy share a common genetic basis^18, 19, 20^ and have a number of neurobiological similarities.^21, 22^ These encompass changes in grey^23, 24^ and white^25, 26^ matter morphology but also neurobiological function,^27, 28, 29, 30^ including cognition.^24, 31, 32, 33, 34^ Various lines of evidence point to a relative weakness of frontal inhibitory mechanisms being a key unifying feature of the schizophrenia spectrum nervous system, a phenomenon described in patients as far back as Bleuler.^35^ In schizotypy this manifests itself in impaired performance on inhibitory tasks (for example, negative priming (NP),^36, 37^ lateral inhibition^38^ and the anti-saccade eye-movement task^39, 40^) as well as working memory (WM) tests^33, 41, 42, 43, 44^ where inhibition of competing stimuli is crucial for successfully maintaining and manipulating information ‘online'. Fine motor control is another neurocognitive function dependent on frontal input that defines schizophrenia spectrum: schizotypal individuals,^45, 46^ as well as patients^47^ have difficulties performing tasks such as smooth eye pursuit where the eyes are guided to follow a moving target. Despite these similarities HS are generally spared psychotic episodes, repeated hospitalisations and chronic antipsychotic treatment.^48^ Importantly, for cognitive experiments intelligence quotient (IQ) and educational levels are within the normal population range.^49^ The combination of schizophrenia spectrum neurobiology and lack of confounds makes HS important surrogates for establishing early evidence of cognitive enhancer efficacy.^10, 11^

Standardised questionnaires such as the Schizotypal Personality Questionnaire (SPQ),^50^ can be used to identify schizotypy psychometrically in the general population.^51, 52^ While the SPQ was designed to identify Schizotypal Personality Disorder (SPD) according to DSM-III-R, in practice high SPQ scorers tend to represent a broader schizotypy group with only ~55% of them attracting a diagnosis of SPD.^50^ A number of other scales are available^53, 54, 55, 56, 57, 58^ but the SPQ is preferred due to the well-established normative data.^59, 60^ A methodological issue with psychometrically identified schizotypy, however, is that cognitive dysfunction may not be readily demonstrable as a meta-analysis in college students reported.^61^ This is an important pragmatic consideration as experimental medicine studies depend on reliable, well defined group differences in performance to merit inclusion in the drug development process.^62, 63^ A potential route towards maximising the potential return on investment in an experimental medicine approach is to investigate whether a particular comparator group yields stronger effects in comparisons with schizotypes. To date large schizotypy studies have focussed on recruiting subjects with average schizotypy scores as comparators reasoning that individuals with low scores may exhibit atypical performance on cognitive tasks.^61, 62^ This reflects the existence of two alternative theories describing the relationship between schizotypy and schizophrenia. A ‘quasi-dimensional' framework proposes that ~10% of the general population have key neural changes (‘schizotaxia') that manifest in the development of schizophrenia or extreme schizotypal symptoms.^17, 64^ According to this model, there is no difference in the neurocognitive performance of healthy volunteers with the exception of individuals with extreme schizotypal symptoms. In contrast a ‘fully dimensional' framework proposes that schizotypal personality traits and its associated neurocognitive changes exist on a continuum from healthy individuals to patients with schizophrenia.^65, 66^ According to this model, average schizotypes (AS) have an intermediate cognitive performance relative to high and low schizotypes (HS and LS).

In the present study, we aimed to establish whether individuals with low or average schizotypy should be used as comparators in studies evaluating putative cognitive enhancers in HS. Thus, we compared the cognitive performance of low, average and HS on a range of cognitive measures (testing inhibitory function and motor control). Based on recent evidence supporting the fully dimensional framework of schizotypy,^67, 68, 69^ we hypothesised that LS would be the comparator group that would yield the more robust statistical difference in comparison with HS.

## Materials and methods

### Subjects, study criteria and design

The study included three groups of participants based on their schizotypy scores as measured by the SPQ (HS, AS and LS defined as scores of >41, 21–36 and ⩽9, respectively). SPQ was chosen as pilot studies had confirmed the sensitivity of SPQ to the factors of interest and enabled us to power the present study to detect significant results.^59^ The HS and AS were recruited within a larger, multi-center study investigating the effects of risperidone, amisulpride and nicotine on cognition in schizotypy. LS were recruited specifically for the purposes of the current analysis.

As part of the procedures of the larger study, AS and HS were recruited from three sites in the United Kingdom (Manchester, London and Cardiff) via an online version of the SPQ in its short^70^ and full version.^50^ The online questionnaire was advertised via university research volunteering e-mails, social networking sites and advertisements in local media. Participants were screened by telephone for significant mental health and medical history. Included participants were invited to a screening appointment at which consent was obtained and the full SPQ was completed again (regardless of whether they had completed the short or full SPQ online). Participants were assigned to the AS or HS groups on the basis of the screening visit full SPQ score only. Other inclusion criteria at the screening visit were age between 18 and 45 years, fluency in the English language, no relevant medical history and body mass index in the range of 18–30. Exclusion criteria were history or presence of mental health disorders (screened for using the Mini-International Neuropsychiatric Interview^71^), daily consumption of more than five cigarettes or eight standard caffeinated drinks, history of migraines, significant visual or hearing impairment. Participants meeting the inclusion criteria were invited to a separate day of testing. On the day, following randomisation, a placebo or nicotine (7 mg) patch was applied to the participants' forearm. Three hours later, a capsule of either amisulpride (400 mg), risperidone (2 mg) or placebo was administered. Approximately 1.5 h later, the participants were tested using a battery of neuropsychological and eye-movement tasks. It consisted of (i) two WM tests (a verbal one and a non-verbal one) based on robust evidence for WM impairment in the schizophrenia spectrum;^33, 41, 42, 43, 44^ (ii) a verbal fluency (VF) task based on the observation that frontal cortex abnormalities are preferentially affected in the schizophrenia spectrum;^27, 28, 36, 37, 72^ (iii) an anti-saccade eye-movement task on the basis of it tapping inhibitory mechanisms and evidence for abnormality in the schizophrenia spectrum;^39, 72, 73^ (iv) a smooth-pursuit eye-movement (SPEM) task on the basis of consistent evidence for smooth-pursuit abnormalities in schizophrenia and schizotypy.^45, 46, 47^ The current study included only participants that were treated with both a placebo patch and a placebo capsule (27 HS and 31 AS). Full details of the study from which these subgroups were selected are detailed in previous publications.^62, 63^

A group of LS was recruited in Manchester only using the online database from the three-centre study described above. Participants attended a single appointment where they provided consent, completed the SPQ and were included if their score was ⩽9 and if they met the same inclusion and exclusion criteria as described above. Included participants completed the same testing battery as the AS and HS groups, but received no treatment. About 35 participants were recruited of which 5 were excluded (4 due to SPQ>9 and 1 participant for having a relevant medical history). Participant demographics are presented in Table 1.

### Task descriptions

#### WM (*N*-Back) task

Series of letters (measuring 4x4 visual degrees) were presented centrally on a monitor. Each letter remained on the screen for 1 s and a blank screen of the same duration separated the stimuli. Participants were instructed to respond by pressing a key when they saw a letter identical to a preceding one with a varying number of letters between the two target letters. The two targets followed each other in the 1-Back condition; they were separated by one and two letters in the 2-Back and 3-Back conditions respectively. There were also blocks to control attention to the task where participants needed to respond when they saw a target letter (0-Back). Before starting the task, participants completed a practice run (one block of each condition). During the task, 3 blocks of each condition were completed (0-Back, 1-Back, 2-Back and 3-Back) with the 12 blocks presented pseudo-randomly. In each block 14 stimuli were presented pseudo-randomly, so that there were 3 target and 11 non-target images. Overall, for every WM load there was a maximum of 9 correct answers and 33 opportunities for errors of commission (that is, opportunities to respond to an incorrect stimulus). For the purposes of statistical analysis, we extracted (i) percentage of correct responses and (ii) number of errors of commission for the 1-Back, 2-Back and 3-Back conditions, yielding a total of six variables.

#### Spatial WM task

A number of treasure chests were presented on a monitor against a background and responses were recorded using a computer mouse. The number of coins and chests were equal in each trial with only one chest containing a coin at any one time. Participants searched for all available coins by clicking on the chests and were instructed not to choose targets they had already found coins. A practice run with three chests was completed, followed by three blocks of four, six and eight treasure chests, respectively (four task repetitions within each block). Between each repetition, the configuration of the boxes on the page was altered so that the likelihood of stereotypical searching strategies was reduced. The outcome variables for each difficulty level (four, six and eight chests) were: (i) average number of within trial errors (selecting a chest previously searched within that trial) and (ii) average number of between trial errors (selecting a chest previously searched within that task repetition) or a total of six variables.

#### VF task

Letter and category VF were assessed during a succession of 1-min periods. In the letter VF task test, participants were asked to name as many words as they could beginning with the letters F, A and S (FAS condition). Names, places or numbers, as well as repetitions were categorised as errors. In the category swap VF test, participants were asked to come up with words from two categories (fruit and furniture) while alternating between them. Only words consistent with the respective categories were recorded as correct. The outcome variables for this task were (i) mean number of correct words across the FAS condition, (ii) number of correct transitions in the category swap condition. Total number of VF variables was 2.

#### Oculomotor tasks

##### Recording

Eye movements were recorded at 1000 Hz sampling rate (EyeLink 1000, SR Research, Kanata, ON, Canada). Participants were seated 57 cm away from a 17-inch monitor with their head resting on a chinrest. The target was a 0.3° diameter black dot presented on a light grey background.

##### Anti-saccade task

A nine-point calibration and a practice trial were carried out before the beginning of the task. A trial started with the black dot in the central position of the screen (0°) for a random duration of 1000–2000 ms. The target then jumped to one of four possible peripheral positions (±7.25°, ±14.5°) for 1000 ms. Each of the possible locations was used 15 times in a random order resulting in 60 trials in total. Participants were required to look at the target in the centre and then to direct their gaze at the exact mirror image position as fast and accurately as possible when the target jumped to either side.

##### SPEM task

In the SPEM task, the target moved horizontally across the screen (from −14.5° to +14.5°) in a sinusoidal waveform at three different target velocities (0.25, 0.5 and 0.75 Hz). It started its movement from −14.5°. For each of the target velocities, the target completed 10 full cycles. Participants were asked to keep their eyes on the target as closely as possible.

##### Analysis

Anti-saccade data were analysed using EyeLink DataViewer software (SR Research). Saccades were defined as having minimum amplitude of 1° and minimum latency of 100 ms. Three variables were extracted from the data: (i) saccade latency; (ii) directional error rate describing the percentage of error trials (that is, trials where the participant's first saccade was towards the target) divided by the total number of valid trials (that is, error trials plus correct trials, excluding eye blink trials); (iii) saccade amplitude.

Two dependent variables were derived for the SPEM analysis using LabView (National Instruments Corporation, Austin, TX, USA) for each of the three speeds (six variables in total). Mean eye velocity was divided by target velocity to yield time-weighted average velocity gains. The analysis included sections in the central 50% of the ramp excluding saccades and eye blink. Saccadic frequency (*N* per second) for each velocity was also measured across the entire pursuit task at each velocity.

### Intelligence quotient

IQ was determined using the National Adult Reading Test,^74^ a reading-based estimate of premorbid intelligence. Participants were asked to pronounce irregularly spelt words from a standardised written list. Individual IQ scores were calculated on the basis of the number of correctly pronounced words.

### Statistical analysis demographic variables

The three groups were compared in terms of their demographic variables (age; premorbid IQ (NART); years of education) and SPQ scores using one-way analyses of variance.

### Statistical analysis test battery

Research aiming to identify biomarkers of disorder by examining cognitive performance on a battery of tests yields a number of dependent variables, often as a function of the number of experimental conditions/stimuli manipulations.

In such exploratory studies, this yields a large number (6 × 30=180) of potential associations that very often exceeds the number of participants. Traditionally, this problem is addressed by choosing a candidate univariate-dependent variable and relevant predictors and a model is constructed using multiple-variable regression. This process is repeated for all dependent variables of interest, leading to the potential problem of accounting for Type II ‘false discovery' errors at the hypothesis testing stage.^75, 76^

An alternative approach is to use data-driven multivariate methods such as principal component analysis to expose the latent structure in a single set of multivariate data, by finding a set of basis vectors onto which observations are projected. This yields a lower dimensional—more parsimonious—representation that explains the largest amount of variance in the data. While this may reduce the number of regression models required, it is agnostic to relationships between the predictors and measurements and there is no *a priori* guarantee that the reduced set of variables will be significantly smaller than the original.

To address these potential issues, we used a two-stage approach. There were two sets of multivariate data, X and Y, representing sets of all observations of the *P*=6 predictors (schizotypy group, study site, sex, years of education, predicted IQ score and age) and *q*=23 candidate biomarkers, respectively. Rather than search for separate reduced-dimensional representations of X and Y independently (that is, using principal component analysis), we first used canonical correlation analysis (CCA)^77^ combined with relevance network detection to extract relationships of interest.^78^ CCA is a technique whereby two sets of multivariate data (in this context, representing multiple demographic and clinical predictors and multiple cognitive battery testing outcomes) can be simultaneously reduced in dimension, exposing any latent structure while maintaining the maximum correlation between the reduced-dimensionality representations. The standard linear regression model is a special case of this method.^77^ The advantage of CCA is that dimensionality reduction of both X and Y is performed simultaneously while preserving maximal correlations between observations of X and Y projected onto these reduced representations. The CCA algorithm is applied iteratively, so that the first application—the leading canonical variates—represents linear combinations of observations in Y best predicted by linear combinations of observations in X. Subsequent applications (revealing the trailing canonical variates) are less predictive and can be discarded because they explain less of the data; analogous to extracting principle components by only keeping the largest eigenvectors.^79^

The computed canonical variates of X and Y are labelled *U*^*L*^ and *V*^*L*^, respectively, with *L*=[1, 2, 3, …, min(*p, q*)]. We chose the first two or three of these canonical variates and then computed another set of equiangular vectors between *U*^*L*^ and *V*^*L*^ such that *Z*^*L*^=*U*^*L*^+*V*^*L*^. Each observation in X and Y was then projected onto *Z*^*L*^ (by inner products), which is equivalent to a correlation measure between each observation in X and Y with the vectors *Z*. Then a similarity score—representing association—was computed by summing over these projections.^78^ These scores were then visualised showing a network of variates in X and Y with the similarity scores representing association strength. By adjusting a threshold *T*=[0,1] over these scores, the visualised network is made simpler or more complex by only showing higher association (as *T* approaches unity) or by allowing more liberal thresholds (as *T* approaches zero), respectively. Those variables which appear in the relevance network are then candidates for further analysis in this instance using regression analyses. To illustrate the effect of the ordinal variables on the biomarker variables, contrasts were computed using Tukey's Honest Significant Difference (HSD) test for multiple comparisons.

### Ethical approval

The study was approved by The University of Manchester Ethics committee (reference number 08176) and carried out in accordance with the ethical standards laid down in the 1964 Declaration of Helsinki.

## Results

### Demographics

The three groups did not differ significantly in terms of age (F(2,85)=1.454, *P*=0.24), IQ (F(2,85)=0.043, *P*=0.96) and gender (LS group 13 f and 14 m; AS group 16 f and 15 m; HS group 15f and 15 m; *χ*2(2)=0.026, *P*=0.98). The years of education model was statistically significant (F(2,85)=5.949, *P*=0.004), which was due to the LS group having significantly more years of education relative to the AS group but not the HS group (Table 1).

### Cognitive tests analysis

We conducted the exploratory CCA on all six demographic variables. Of the measured biomarker variables, we removed those which were of no interest, that is, cognitive performance at the control (‘0-Back') condition of the *N*-Back task. To avoid difficulties with matrix calculations and data imputation involved in CCA, we only included participants with complete data sets on all demographic and biomarker variables, leading to the inclusion of *n*=83 participants to derive the relevance network (five LS participants excluded). The final sample size was LS 22, AS 31 and HS 30.

**Figure 1:**
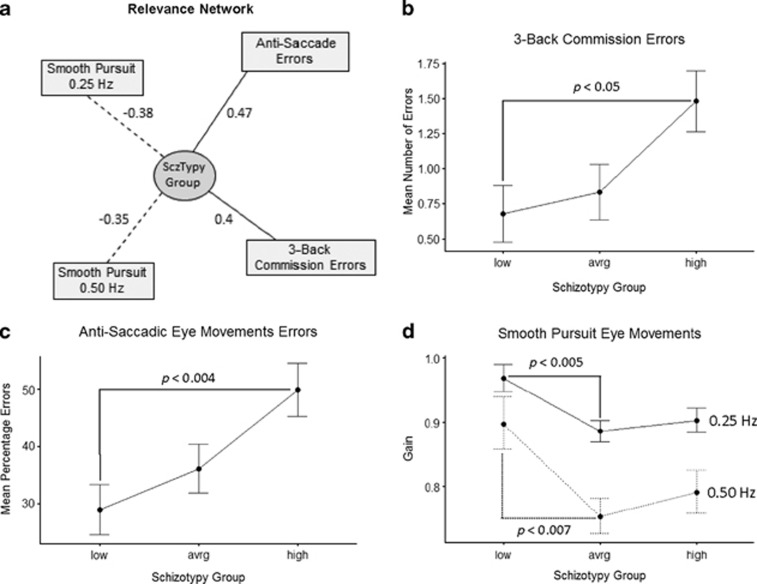
(**a**) Relevance network extracted from complete data set (dotted lines—negative correlation between variables; solid lines—positive correlation between variables; numbers indicate direction and strength of association); corresponding generalised linear models (GLMs) for ordinal schizotypy group predicting mean number of commission errors on 3-Back task; (**b**) mean percentage errors for anti-saccade task; (**c**) mean pursuit velocity gain (° per second) at target velocities of 0.25 and 0.5 Hz; (**d**) significant multiple general linear hypothesis tests (Tukey contrasts) for low, average and high schizotypy are show with *P*-values for each GLM.

Using the first *L*=[1,2,3] canonical variates and a liberal threshold *T*=0.35, we were able to derive a relevance network, which revealed associations that might be usefully subjected to regression analyses to quantify association of a given demographic variable (in X) with its associated biomarker (in Y) shown in Figure 1a.

Of all the demographic variables (schizotypy group, study site, sex, years of education, predicted IQ score and age), in X, schizotypy group was the only variable showing associations in the relevance network. Years of education had relatively weak associations, which did not survive the T threshold. For the candidate biomarkers, Y, the schizotypy group relevance network showed negative associations for velocity gain on the (i) 0.25 Hz and (ii) 0.50 Hz sinusoid smooth-pursuit task and positive associations between (iii) mean percentage errors on the oculomotor anti-saccade task and (iv) mean number of commission errors on the 3-Back WM task. The relevance network did not demonstrate significant associations between schizotypy and the Spatial WM or VF variables.

To explore the quantitative relationship between the relevant variables, generalised linear models were constructed for schizotypy group (as an ordinal variable) and the 3-Back commission errors (Figure 1b), the mean percentage errors on the anti-saccade task (Figure 1c) and the ratio of velocity gain scores for the two SPEM variables (Figure 1d). To illustrate the effect of schizotypy group (low, average and high) on the biomarker variables, contrasts were computed using Tukey's HSD for multiple comparisons (Table 2). The results confirmed the observed main effect of schizotypy group and demonstrated that LS vs HS comparisons were more consistently statistically significant relative to AS vs HS comparisons. With respect to the anti-saccade error rate and *N*-Back errors of commission, the LS vs HS comparison was statistically significant while the AS vs HS comparison approached significance. In the SPEM models, the LS group performed significantly better than the AS group and the LS vs HS comparisons were significant at trend level. In contrast there was no significant difference between AS and HS groups in terms of SPEM performance.

## Discussion

The objective of this study was to clarify the relationship between levels of schizotypy and neurocognition by comparing the cognitive performance of three groups of healthy volunteers defined by their SPQ scores. Participants were assessed on a range of neuropsychological and eye-movement tests that are abnormal across the schizophrenia spectrum to establish whether LS or AS is the optimal control group for comparison with HS in translational studies of cognitive enhancers.

Of the candidate biomarker variables in the five tasks, those that measured inhibitory control and had sufficient difficulty, were most sensitive to schizotypy. For example, in *N*-Back, only errors of commission (dependent on the ability to inhibit responses to irrelevant stimuli) at the most difficult condition (3-Back) showed HS performing worse than LS. In contrast, the groups did not differ in percentage of correctly identified *N*-Back stimuli or any spatial WM variables. Similarly, in the anti-saccade task (where inhibitory control is crucial) HS made significantly more errors relative to LS and AS. In the SPEM task, only the 0.25 and 0.50 Hz conditions were sensitive to schizotypy levels, but importantly this effect was evident only in comparisons between LS and HS. In the 0.75 Hz condition, the groups did not significantly differ suggesting that all participants found this condition difficult. The follow-up regression models confirmed that for each of the four variables, where the CCA model identified a schizotypy effect, the LS vs HS comparisons showed more robust and significant effects relative to AS vs HS (Table 2).

These data indicate that differences in performance on cognitive and oculomotor tasks are largest between LS and HS groups. This is consistent with the ‘fully dimensional' model of schizotypy,^65^ which has recently been supported by convergent lines of evidence.^80^ For example, the latent structure of schizotypy personality features were found to fit a dimensional distribution in a taxomeric analysis.^69^ In addition, psychotic-like experiences (hallucinations and delusional ideation) in the general population fit a continuous distribution.^67, 68^ These findings are of particular relevance to translational studies seeking evidence for cognitive enhancer efficacy, that is, enrichment of schizotypy studies is best achieved by comparing LS and HS groups while using tasks that maximise the extent of cognitive difference between these groups. The potential utility of such enrichment is highlighted by our finding that on a number of measures HS were indistinguishable from the control groups. This argues that psychometrically defined schizotypes are a high functioning population, a conclusion consistent with the practice of schizotypy studies recruiting participants among university and college students. Also while some studies do not find difference in cognition between HS and controls (either LS or AS) abnormal functional imaging response is demonstrable.^21^ This demonstrates the risks involved in using a single task to assess cognition in this high functioning group. A popular approach in schizophrenia^6^ and Alzheimer's disease^81^ research, and adopted for this study, is to use a battery of tests.^62, 63, 82^ However, this strategy risks increasing the false positive rate through multiple comparisons.^75, 76^ We sought to address this issue by performing a canonical analysis^77^ which identified the variables where a significant effect of schizotypy was present thereby greatly reducing the number of *post hoc* comparisons. We propose that similar analyses should be used in the initial work-up of battery data sets.

The finding of preferential impairment in measures of inhibitory processes is consistent with evidence in schizotypy,^22^ while inhibitory control deficits have been proposed as core features of schizophrenia.^35^ Several tests have been developed to probe inhibition directly (for example, NP and lateral inhibition tasks). In NP, participants are instructed to attend to previously inhibited distractors. Normal inhibitory function leads to increased reaction times to the previously ignored stimulus. In both patients with schizophrenia and schizotypes, this delay is diminished.^36, 37, 83^ Lateral inhibition is based on the observation that conditioning to a stimulus is slower if it has previously been presented without consequence. Similar to NP, the period of conditioning is shortened in schizophrenia patients^84^ and schizotypes.^72, 85, 86^ Successful inhibition is critical for complex cognitive functions such as WM, which has emerged as a potential endophenotype of schizophrenia.^87^ This is based on studies reporting subtle WM impairment in individuals with genetic^88, 89^ and phenomenological^90, 91^ vulnerability to schizophrenia. The currently reported *N*-Back performance in schizotypes adds to a growing body of evidence showing WM impairment in schizotypy.^33, 41, 44^ Our data also highlight that not all WM tasks are sensitive to schizotypy. Instead it appears that elements directly relevant to inhibition are more useful in this high functioning group.

Failure of the HS group to inhibit saccades in the anti-saccade task also replicates previous findings.^39, 40, 46, 73, 92^ This abnormality is again shared between HS and schizophrenia patients^47^ and was recently shown to be dependent on a neural circuitry encompassing putamen, thalamus, cerebellum and visual cortex.^72^ We also found that saccade gain during SPEM was lower in HS, a finding replicating previous studies.^45, 46^ The ability to follow a visual object moving at constant speed depends on a fronto-parieto-occipital network, which projects an expected target trajectory to the motor cortex.^93^ The generated prediction is then continuously corrected with occipital cortex tracking data. Evidence regarding the neural basis of smooth-pursuit impairment in schizotypy comes from a fMRI study demonstrating lower BOLD occipital signal.^94^ This finding is compatible with reports of abnormal occipitally generated evoked responses in schizotypy^32^ and schizophrenia.^95, 96, 97^ It is known that sensory cortex is under frontal cortex modulatory control^98^ and it may be that a primary dysfunction in these afferents underlies the observed occipital deficit.^99^

### Limitations

There are several limitations of this exploratory analysis. Firstly, the AS and HS groups were recruited as part of a larger placebo-controlled study which investigated the effects of acute administration of risperidone, amisulpride and nicotine on cognition. AS and HS participants were required to attend two appointments (treatment was given, and data on treatment effects on cognitive performance were obtained, on the second, full-day appointment), were placebo-treated and were recruited from three sites in the UK. In contrast, the LS group attended a single 3-h appointment in which the data were recorded, did not receive placebo medication and were recruited in Manchester only. We therefore cannot rule out the possibility that the superior performance of the LS group was due to these protocol differences. It is feasible that fatigue or placebo effects could have led to reduced performance in AS relative to LS groups. However, the superior performance of the LS group was confined solely to measures where a difference between the placebo-treated AS and HS groups was already evident and for which compelling evidence for an abnormality in the schizophrenia spectrum already exists. The lack of schizotypy effect on other tasks argues against a generalised placebo or fatigue effects-driven cognitive superiority in the LS group. Secondly, AS group had a significantly lower number of years of education compared with the LS group. We accounted for this significant difference by including years of education as a predictor in the canonical testing and the results showed that this variable did not have a significant independent effect on the biomarker variables. In addition, the groups did not differ in terms of projected IQ, a measure arguably more relevant to cognition research than years of education which tends to reflect socio-economic status. Finally, we did not have information on the family history of schizophrenia among the participants—it is possible that the groups were different in this respect.

## Conclusion

In summary, this study explored the neurocognitive correlates of low, average and high schizotypy. The results revealed an impairment that primarily affected inhibitory processes in HS subjects. Importantly we found that the performance of AS subjects in these tests was intermediate between that of LS and HS subjects. These results argue in favour of the fully dimensional theory of schizotypy and strongly suggest that subjects with LS are the most appropriate control group for further experimental medicine schizotypy studies with putative cognition enhancing agents for the treatment of cognitive deficits in schizophrenia.

## Figures and Tables

**Table 1:** Summary of the GLMs

*Variable*	*Schizotypy group contrast*	*Estimate/coefficient*	*s.e.*	P*-value*
Years of education	High vs average	0.808	0.572	0.162
	**Low** vs **average**	**1.915**	**0.557**	**0.001**
	Low vs high	1.107	0.577	0.058
Smooth pursuit velocity gain (0.25 Hz)	High vs average	−0.021	0.032	0.78
	**Low** vs **average**	−**0.096**	**0.031**	**0.005**
	Low vs high	−0.075	0.032	0.053
Smooth pursuit velocity gain (0.50 Hz)	High vs average	−0.063	0.072	0.654
	**Low**vs **average**	−**0.213**	**0.070**	**0.006**
	Low vs high	−0.150	0.073	0.099
Anti-saccade mean percentage errors	High vs average	13.676	6.266	0.074
	Low vs average	−7.169	6.080	0.466
	**Low vs high**	−**20.844**	**6.366**	**0.003**
3-Back mean number of commission errors	High vs average	0.647	0.291	0.068
	Low vs average	−0.155	0.283	0.848
	**Low vs high**	−**0.801**	**0.296**	**0.019**

Abbreviation: GLM, generalised linear model.

GLMs were constructed for each relevant variable from the canonical correlation analysis, with the three-level ordinal variable schizotypy group predicting the measured task performance variable. Contrast estimates for relative levels of schizotypy (rather than beta coefficients) and s.e. are shown.

Significant results are shown highlighted in bold for confidence level *P*<0.05.

**Table 2:** Demographics and descriptives

*Variable*	*Low schizotypy*		*Average schizotypy*		*High schizotypy*	
	*Mean*	*s.d.*	*Mean*	*s.d.*	*Mean*	*s.d.*
*Demographics*						
SPQ score	3.8	3.1	27.8	4	50.1	6.1
Age	25.3	5.0	23.8	4.6	25.4	5.8
Premorbid IQ (NART)	114.2	6.2	114.2	4.9	113.8	6.0
Years of education	16.3	2.2	15.5	1.7	17.4	2.6
						
N-*Back* *percentage correct*						
1-Back	99.6	0.8	99.5	0.7	97.8	0.8
2-Back	92.2	2.6	96.2	2.5	94.7	2.8
3-Back	86.7	2.3	83.3	2.3	78.1	2.5
						
N*-Back* *errors of commission*						
1-Back	0.1	0.1	0.1	0.1	0.3	0.1
2-Back	0.1	0.1	0.0	0.1	0.2	0.1
3-Back	0.1	0.2	0.7	0.2	1.4	0.2
						
*Spatial working memory between search errors*						
Load 1	0.1	0.1	0.1	0.1	0.0	0.1
Load 2	0.7	0.2	0.5	0.2	0.5	0.2
Load 3	1.0	0.4	2.0	0.4	2.0	0.4
						
*Spatial working memory: within search errors*						
Load 1	0.0	0.0	0.1	0.0	0.0	0.0
Load 2	0.2	0.1	0.1	0.1	0.3	0.1
Load 3	0.6	0.3	0.5	0.3	0.4	0.3
						
*Verbal fluency: mean number correct words*						
FAS task	14.6	4.0	15.5	4.3	13.7	3.6
Category swap task	7.7	1.4	8.0	1.5	7.1	1.3
						
*Verbal fluency: mean number correct transitions*						
Category swap task	6.8	0.3	7.7	0.3	7.4	0.3
						
*Anti-saccade*						
Errors	28.2	17.1	36.2	24.2	49.5	26.3
Amplitude gain	119.4	23.5	122.2	28.0	116.1	37.1
Peak velocity	295.8	47.3	302.3	70.9	298.4	63.4
						
*Smooth pursuit eye-movement task: velocity gain*						
0.25 Hz velocity	97.1	4.7	89.9	10.2	91.24	8.7
0.50 Hz velocity	84.2	14.4	78.9	15.9	82.3	14.4
0.75 Hz velocity	67.4	20.7	61.1	23.2	67.3	20.1
						
*Smooth pursuit eye-movement task: saccadic frequency*						
0.25 Hz velocity	0.8	0.4	0.9	0.4	0.8	0.3
0.50 Hz velocity	1.4	0.4	1.5	0.4	1.6	0.5
0.75 Hz velocity	2.3	0.5	2.2	0.6	2.5	0.7

Abbreviations: FAS task (a verbal fluency task - see subjects and methods); NART, national adult reading test; SPQ, schizotypal personality questionnaire.
